# Supplementary material for: Personalized Clostridioides difficile engraftment risk prediction and probiotic therapy assessment in the human gut
Source: bioRxiv. 2024 Jan 4:2023.04.28.538771. Originally published 2023 Apr 29. Preprint. [Version 3] doi: 10.1101/2023.04.28.538771 (PMC10168307; doi:10.1101/2023.04.28.538771)
Supplement: 1 [file NIHPP2023.04.28.538771v3-supplement-1.pdf]

bioRxiv preprint doi: https://doi.org/10.1101/2023.04.28.538771; this version posted January 4, 2024. The copyright holder for this preprint (which was not certified by peer review) is the author/funder, who has granted bioRxiv a license to display the preprint in perpetuity. It is made available under aCC-BY 4.0 International license.

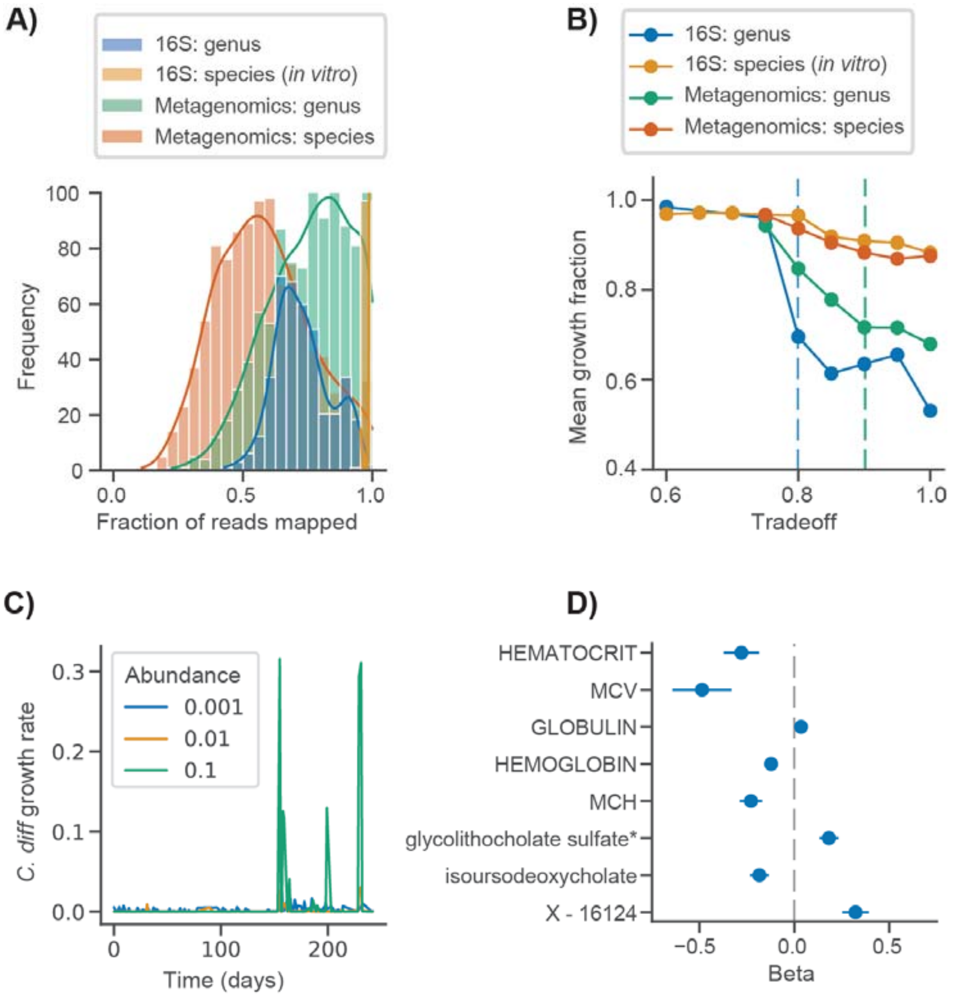

Figure S1. Development of *in silico C. difficile* invasion assay. (**A**) Histograms displaying the fraction of reads mapped at the genus and species levels for the David *et al.* and Hromade *et al.* 16S amplicon data and Ianiro *et al.* metagenomics data using an NCBI reference and metabolic models from the AGORA database. (**B**) Mean growth fraction across samples, datasets, and taxonomic mappings (e.g., fraction of taxa with estimated growth rate $>10^{-6}$) as a function of model tradeoff value. Dashed lines indicated the tradeoff values chosen for subsequent analyses. The blue dashed line indicates the value used for 16S data and green dashed line denotes the value used for metagenomics data. Tradeoff values were chosen such that mean growth fractions of ~0.7 were achieved using genus level mapping. (**C**) Relationship between *C. difficile*

bioRxiv preprint doi: https://doi.org/10.1101/2023.04.28.538771; this version posted January 4, 2024. The copyright holder for this preprint (which was not certified by peer review) is the author/funder, who has granted bioRxiv a license to display the preprint in perpetuity. It is made available under aCC-BY 4.0 International license.

invasion abundance and growth rate for one of the two David *et al.* time series. (**D**) Association coefficients for estimated *C. difficile* log growth rate, blood metabolite concentrations, and clinical labs for the Arivale cohort.

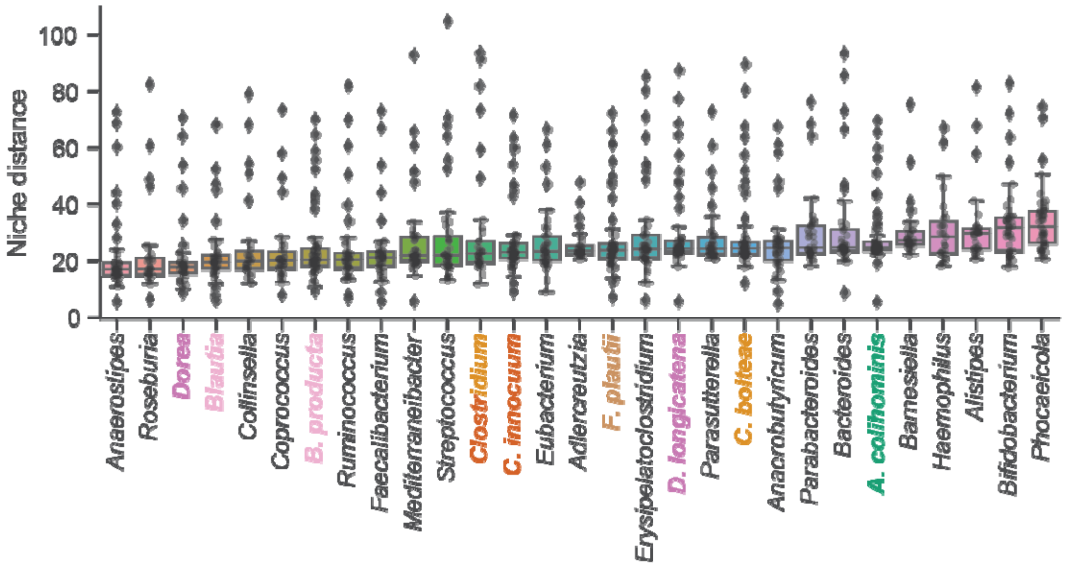

Figure S2. Probiotic strains and associated genera have niche distances close to *C. difficile* relative to unrelated genera. Niche distances of strains and genera, represented as the euclidean distance between flux vectors, relative to *C. difficile* across CDI-FMT cohort samples for which the *C. difficile* growth rate $>10^{-6}$. Genera and strains are ordered by the median niche distance. Probiotic strains and associated genera are colored, consistent with the legend in Fig. 5D. *C. bolteae* and *C. innocuum* are both members of the genus *Clostridium*.
