# Supplementary material for: Personalized Clostridioides difficile engraftment risk prediction and probiotic therapy assessment in the human gut
Source: bioRxiv. 2023 May 9:2023.04.28.538771. Originally published 2023 Apr 29. Preprint. [Version 2] doi: 10.1101/2023.04.28.538771 (PMC10168307; doi:10.1101/2023.04.28.538771)
Supplement: Supplementary file 1 [file NIHPP2023.04.28.538771V2-supplement-1.pdf]

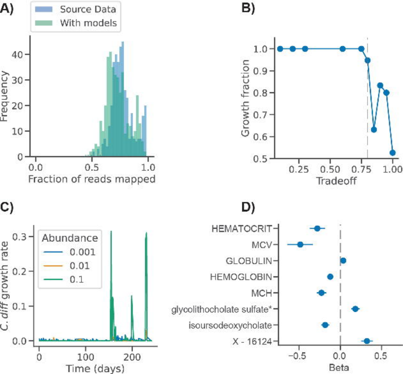

A)
Source Data
With models
Frequency
Fraction of reads mapped
B)
Growth fraction
Tradeoff
C)
Abundance
0.001
0.01
0.1
C. diff growth rate
Time (days)
D)
HEMATOCRIT
MCV
GLOBULIN
HEMOGLOBIN
MCH
glycolithocholate sulfate*
isoursodeoxycholate
X - 16124
Beta

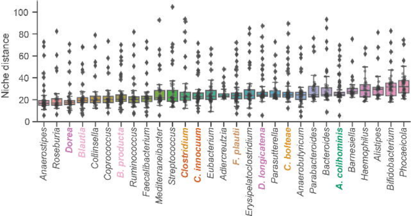

Niche distance
0
20
40
60
80
100
Anaerostipes
Roseburia
Dorea
Blautia
Collinsella
Coprococcus
B. producta
Ruminococcus
Faecalibacterium
Mediterraneibacter
Streptococcus
Clostridium
C. innocuum
Eubacterium
Adlercreutzia
F. plautii
Erysipelatoclostridium
D. longicatena
Parasutterella
C. bolteae
Anaerobutyricum
Parabacteroides
Bacteroides
A. colihominis
Barnesiella
Haemophilus
Alistipes
Bifidobacterium
Phocaeicola
